# Ultrahigh-sensitive optical coherence elastography

**DOI:** 10.1038/s41377-020-0297-9

**Published:** 2020-04-13

**Authors:** Yan Li, Sucbei Moon, Jason J. Chen, Zhikai Zhu, Zhongping Chen

**Affiliations:** 1 Beckman Laser Institute, University of California, Irvine, Irvine, CA 92612 USA; 2 Department of Biomedical Engineering, University of California, Irvine, Irvine, CA 92617 USA; 3 Department of Physics, Kookmin University, Seoul, 02707 South Korea

**Keywords:** Optical techniques, Biophotonics

## Abstract

The phase stability of an optical coherence elastography (OCE) system is the key determining factor for achieving a precise elasticity measurement, and it can be affected by the signal-to-noise ratio (SNR), timing jitters in the signal acquisition process, and fluctuations in the optical path difference (OPD) between the sample and reference arms. In this study, we developed an OCE system based on swept-source optical coherence tomography (SS-OCT) with a common-path configuration (SS-OCE_CP_). Our system has a phase stability of 4.2 mrad without external stabilization or extensive post-processing, such as averaging. This phase stability allows us to detect a displacement as small as ~300 pm. A common-path interferometer was incorporated by integrating a 3-mm wedged window into the SS-OCT system to provide intrinsic compensation for polarization and dispersion mismatch, as well as to minimize phase fluctuations caused by the OPD variation. The wedged window generates two reference signals that produce two OCT images, allowing for averaging to improve the SNR. Furthermore, the electrical components are optimized to minimize the timing jitters and prevent edge collisions by adjusting the delays between the trigger, k-clock, and signal, utilizing a high-speed waveform digitizer, and incorporating a high-bandwidth balanced photodetector. We validated the SS-OCE_CP_ performance in a tissue-mimicking phantom and an in vivo rabbit model, and the results demonstrated a significantly improved phase stability compared to that of the conventional SS-OCE. To the best of our knowledge, we demonstrated the first SS-OCE_CP_ system, which possesses high-phase stability and can be utilized to significantly improve the sensitivity of elastography.

## Incredibly detailed elastic imaging achieved with precision optics

An ultrahigh-sensitive optical method for imaging the elasticity of biological samples can detect vibrations smaller than a nanometer in size. Elastography measures the stiffness of biological tissues, for example distinguishing cancer tumors from healthy tissue, usually via ultrasound elastography or magnetic resonance elastography. More recent “optical coherence elastography” is an non-invasive imaging method can gives more details on the sample’s elastic properties than conventional methods. Yan Li and her co-works at Dr. Zhongping Chen’s research group from the University of California, Irvine have further improved the sensitivity of this method by incorporating two reference beams of different frequencies using a common-path configuration. The produced two OCT images can then be averaged, greatly enhancing the signal-to-noise ratio. The phase stability of their system is high enough to measure displacements as small as 300 pm, and provided detailed elastic images of a live rabbit cornea. This advance is the stepping stone to translate this technology for diagnosis and managment of various ocular diseases involving change in the elastic properties of cornea, retina, and optical nerve head.

## Introduction

Optical coherence elastography (OCE) is an emerging functional imaging technique that quantifies the elasticity of biological tissue by using Doppler optical coherence tomography (OCT) to measure the local tissue displacement as a function of the applied stress^1,2^. Compared with conventional elastography (e.g., magnetic resonance elastography, ultrasound elastography, and Brillouin microscopy), OCE possesses micron-level resolution and axial displacement sensitivity on the order of subnanometres and therefore has become an attractive research tool for ophthalmology, dermatology, cardiology, and oncology^3–9^.

Since OCE relies on the measuring phase via Doppler OCT, the phase stability of the imaging system is the key factor determining its performance^10–12^. Most OCE systems utilizing the acoustic radiation force (ARF) as a tissue excitation method are reported to have the capability of detecting displacements in the range of hundreds of nanometers. In those cases, a relatively strong ARF is necessary to accurately reconstruct the elastic wave propagation, and this force may exceed the ophthalmic mechanical index (MI) safety standard of 0.23 approved by the Food and Drug Administration^13–15^. An OCE system with ultrahigh displacement sensitivity will be able to scale down the applied ARF by at least 1 order of magnitude while maintaining a sufficient signal-to-noise ratio (SNR), which will reduce the required ARF such that it is within the range of the MI safety standard to facilitate the clinical translation of OCE in ophthalmology. The common-path configuration, as an optical method, addresses the phase instability caused by environmental vibration that leads to a fluctuating optical path difference (OPD) between the reference and sample arms. Additionally, the common-path configuration provides intrinsic compensation for polarization mismatch and dispersion mismatch between the sample and reference arms^16–18^. In 2017, Lan et al. reported a high-phase-stability OCE system using a common-path spectral-domain OCT (SD-OCT) to achieve a subnanometre displacement sensitivity, demonstrating the feasibility of common-path OCE; however, only phantom experiments were performed^19^. In addition, SD-OCT-based OCE systems are based on a static operation principle that provides phase-stable detection^15,20–24^, but their performance is limited by the groove density of the diffraction grating, the center wavelength of the light source, and the resolution of the line-scan camera, hence the inherent disadvantages of phase washout, low imaging speed, shallow penetration depth, and short imaging range^25,26^.

In contrast, swept-source optical coherence tomography (SS-OCT), which can be operated with a much narrower instantaneous linewidth, longer center wavelength, higher repetition rate, and balanced detection, has the capability of providing a long imaging range, deep penetration depth, high imaging speed, and reduced fringe washout. Nevertheless, the phase stability of SS-OCT suffers from fluctuations in the mechanical movement of the sweeping laser; thus, a proper synchronization between the data acquisition and laser sweep is crucial in achieving a high-phase stability in a swept-source system^27^. A lambda (λ) trigger using a fiber Bragg grating (FBG) as well as a k-clock generated by a Mach-Zehnder interferometer (MZI) have been introduced to improve the phase stability, making SS-OCT a more attractive setup in OCE applications^28^. In recent years, several OCE systems based on conventional SS-OCT (SS-OCE_COV_) have been proposed^14,29–35^, and their feasibilities have been validated through ex vivo and in vivo experiments, demonstrating great potential towards clinical translation. Although these studies have reported subnanometre displacement sensitivities, this is achieved only in system charaterization where a simple common-path configuration is used. The system performance of SS-OCE_COV_ is degraded when performing measurements because the phase fluctuation between the sample and reference arms is not negligible in conventional OCE. Furthermore, the reported SNR and phase stability are usually from the averages of several measurements^36^. Despite current advancements in SS-OCT, achieving a phase sensitivity on the order of subnanometres from actual experiments using SS-OCE remains challenging.

In our study, we designed and implemented a high-phase-stable OCE system using a common-path SS-OCT (SS-OCE_CP_). A 3-mm thick, 30-arcmin wedged glass window (WW10530, Thorlabs, Inc., NJ) was incorporated distal to the scan lens to generate two reference signals, each from one of the surfaces of the window. This setup allows for the simultaneous generation of two OCT interference signals in two different frequency domains, which can be averaged for an enhanced SNR and reduced speckle. This averaging method is not achievable in SD-OCT due to the limited imaging range. Furthermore, the common-path configuration minimizes the differences between the sample and the reference arm, thereby providing stable phase information for precision displacement measurement. In addition to the optical method, data acquisition and synchronization were optimized to accurately retrieve the phase information. In this report, we first compared the phase performance of SS-OCE_CP_ with that of SS-OCE_COV_. Then, a tissue-mimicking phantom model and an in vivo rabbit model were imaged to validate the SS-OCE_CP_ performance.

## Results

### Phase stability quantification

**Fig. 1:**
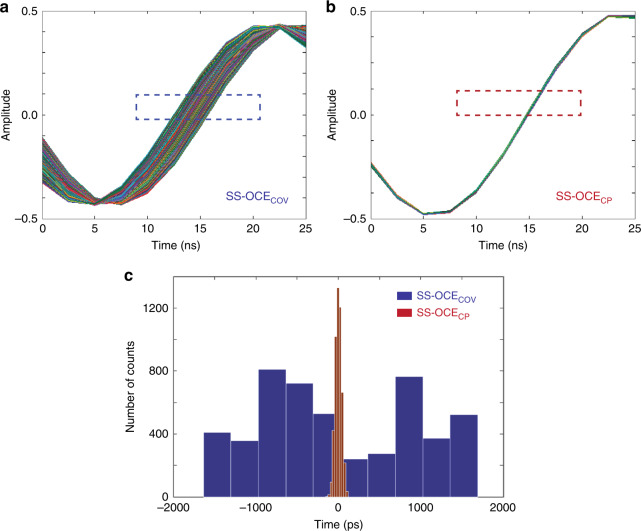
Phase stability quantification. Overlay of 5000 interference fringes obtained using (**a**) SS-OCE_COV_ and (**b**) SS-OCE_CP_. **c** Timing consistency at zero crossings

To measure the phase stability of our SS-OCE_CP_, a 1.0-mm microscope slide was placed at the focus of the objective lens to generate autocorrelation interference by the back-reflected light from the front and back surfaces of the slide. In our SS-OCE_COV_ counterpart, a gold mirror was placed at the focus of the objective lens to generate an OCT interference signal with the same frequency as that of SS-OCE_CP_. The length of the reference arm was adjusted by 1.40 mm to offset the zero OPD. In both cases, 5000 A-lines were acquired sequentially. Figure 1a, b shows the overlaid interference fringes (*n* = 5000) from SS-OCE_COV_ and SS-OCE_CP_, respectively. Temporal shifting is much more severe in SS-OCE_COV_, which implies a better timing stability in SS-OCE_CP_. For the temporal analysis, the timing information of each zero-crossing was obtained through linear interpolation (blue and red dashed boxes in Fig. 1a, b, respectively), and the standard deviation of the timing variation corresponds to the phase stability of the system. For SS-OCE_COV_, the standard deviation was calculated to be 940 ps, whereas that of SS-OCE_CP_ was 35 ps. The corresponding histograms are shown in Fig. 1c. In addition, the phase stabilities of SS-OCE_COV_ and SS-OCE_CP_, which were 175 mrad (~13-nm displacement) and 4.2 mrad (~0.3-nm displacement), respectively, were determined by calculating the standard deviation of the phase angle at the peak in the frequency domain, demonstrating a >40-fold phase stability improvement in SS-OCE_CP_ compared to SS-OCE_COV_.

### Enhanced SNR

**Fig. 2:**
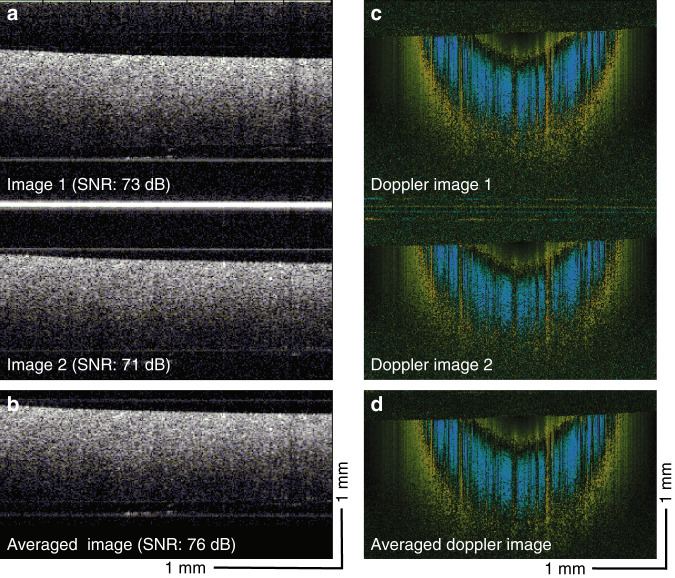
SNR quantification. **a**, **b** Original and averaged OCT images, respectively. **c**, **d** Original and averaged Doppler OCT images, respectively

Since the 3-mm wedged window generates two OCT images in different frequency domains, these two OCT images can be averaged to enhance the SNR, further improving the phase stability. Figure 2a shows the OCT images of the phantom obtained via SS-OCE_CP_; the top and bottom images are the interference signals generated by the back-reflected light from the back and front window surfaces, respectively, with the backscattered light from the phantom. The high-intensity horizontal line between the two OCT images is the interference signal from the two surfaces of the window. The two images are separated by ~3.4 mm. By averaging the two images, the SNR was improved from 73 and 71 to 76 dB, an approximately 3-dB improvement (Fig. 2b). The enhanced SNR through averaging is also reflected on the Doppler OCT images. Figure 2c shows the pair of Doppler images obtained via SS-OCE_CP_, and Fig. 2d shows the averaged result, demonstrating the reduced noise floor and hence improved SNR.

### Phantom experiment

**Fig. 3:**
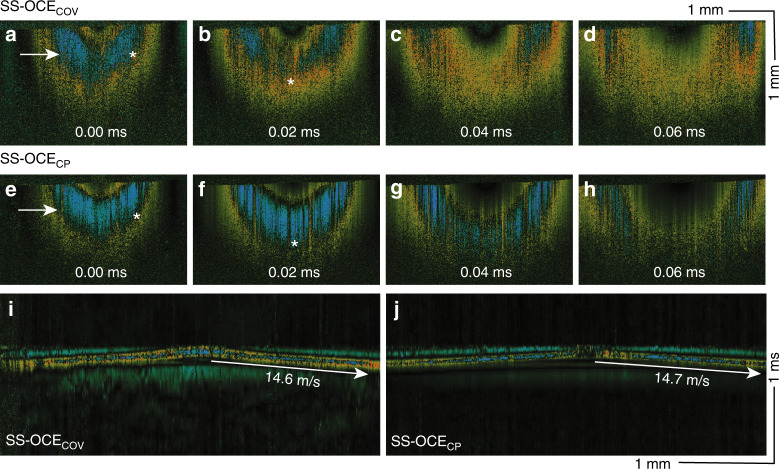
Elastic wave in the silicone phantom. Time-lapse Doppler OCT B-scans obtained using (**a**–**d**) SS-OCE_COV_ and (**e**–**h**) SS-OCE_CP_. **i**, **j** Spatiotemporal Doppler OCT at a depth indicated by the white arrows in (**a**) and (**e**), respectively

After demonstrating the improved SNR and phase stability of the SS-OCE_CP_ system, the elastograms of the phantom obtained using both SS-OCE_COV_ and SS-OCE_CP_ were compared. The time-lapse Doppler images of SS-OCE_COV_ and SS-OCE_CP_ are shown in Fig. 3a–d and Fig. 3e–h, respectively. While an outward propagation of the spherical elastic waves was observed in both cases, the SS-OCE_CP_ result reveals a more pronounced boundary between the upward (yellow) and downward (blue) displacement, denoted by the white * in Fig. 3. In addition, this displacement boundary is better maintained in the deeper region when imaged using SS-OCE_CP_ (same white * in Fig. 3b, f). Since the deeper region of the image is encoded in the higher-frequency components of the interference signal, a higher phase stability is necessary to reveal the detailed information in those regions. This is further exemplified by the less-phase-stable SS-OCE_COV_, in which the displacement boundary is more difficult to demarcate (Fig. 3a–d). Figure 3i, j shows the spatiotemporal images from SS-OCE_COV_ and SS-OCE_CP_ at the depths indicated by the white arrows in Fig. 3a, e, respectively. The significantly reduced noise floor can be visualized in the SS-OCE_CP_ image (Fig. 3j). The propagation speed of the elasticity wave and Young’s modulus of the phantom are shown in Fig. S1.

### **In vivo** rabbit experiment

**Fig. 4:**
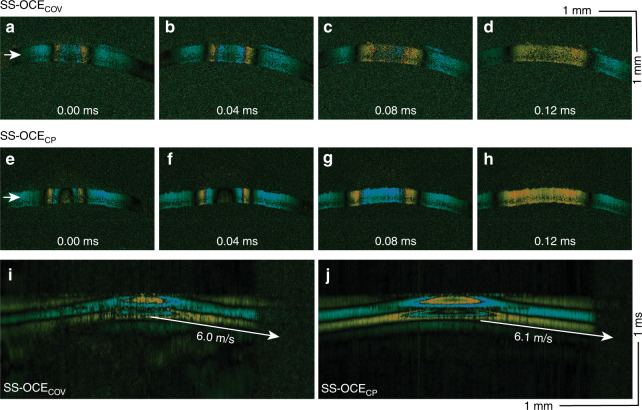
Elastic wave in rabbit cornea. **a**–**d** Time-lapse Doppler OCT B-scans from the SS-OCE_COV_ system. **e**–**h** Time-lapse Doppler OCT B-scans from the common-path OCE system. **i**, **j** Spatiotemporal Doppler OCT at a depth indicated by the white arrows in (a) and (e), respectively

We further verified the performance of the proposed SS-OCE_CP_ in a rabbit model. Figure 4a–d and Fig. 4e–h show the time-lapse Doppler OCT B-scans of rabbit cornea obtained using SS-OCE_COV_ and SS-OCE_CP_, respectively. In these figures, an elastic wave that propagates outwards from the center can be visualized. Figure 4i, j shows the corresponding spatiotemporal images and propagation speeds of two OCE systems at the depths indicated by the white arrows in Fig. 4a, e. The propagation speed of the elasticity wave and Young’s modulus of the cornea are shown in Fig. S1. The results concur with the phantom experiment, demonstrating the enhanced phase stability of SS-OCE_CP_, which allows for a more pronounced displacement boundary, an improved SNR in the deeper region, and reduced background noise.

**Fig. 5:**
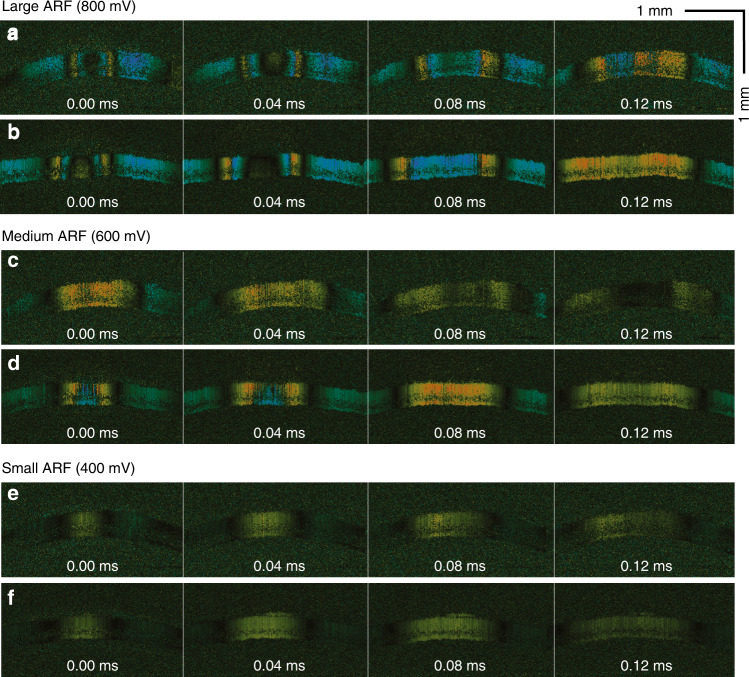
Time-lapse Doppler OCT B-scans from SS-OCE_COV_ and SS-OCE_CP_ systems with different ARFs. **a**, **c**, **e** Time-lapse Doppler OCT B-scans from the SS-OCE_COV_ system with large, medium, and small ARFs, respectively. **b**, **d**, **f** Time-lapse Doppler OCT B-scans from the SS-OCE_CP_ system with large, medium, and small ARFs, respectively

**Fig. 6:**
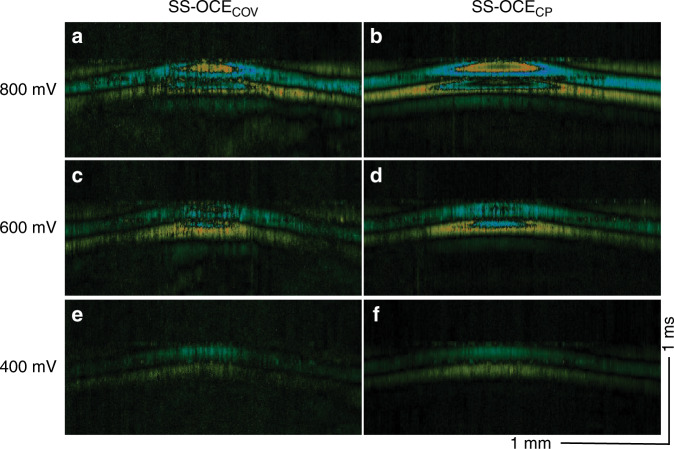
Spatiotemporal Doppler OCT of the cornea for different ARFs. **a**, **c**, **e** B-scans from the SS-OCE_COV_ system excited with large, medium, and small ARFs, respectively. (**b**), (**d**), and (**f**): B-scans from the SS-OCE_CP_ system excited with large, medium, and small ARFs, respectively

A highly phase-stable OCE system can detect smaller displacements with improved accuracy. To investigate the influence of the generated ARF amplitude on the phase information retrieval, we applied three different levels of ARF, from 800 to 400 mV, using the same rabbit cornea model (Fig. 5). In all cases, SS-OCE_CP_ provided a better visualization of elastic wave propagation than did SS-OCE_COV_. In the 800-mV SS-OCE_COV_ case, the phase error can be observed at 0.12 ms in Fig. 5a. When applying 600-mV ARF to SS-OCE_COV_, although bidirectional wave propagation can be identified in the center at 0 ms (Fig. 5d), only one direction of displacement was observed at 0.04 ms (Fig. 5c). A moderately poorer SNR was also observed in the 400-mV SS-OCE_COV_ (Figs. 5e versus f). The corresponding spatiotemporal Doppler OCT images are shown in Fig. 6.

Last, we tested the change in elastic wave velocity in rabbit corneas in vivo with normal and high intraocular pressure (IOP) to further verify the capability of SS-OCE_CP_. A positive correlation between corneal elasticity and IOP was previously reported, and our results (Supplementary Material, Figs S2 and S3), demonstrating the same correlation, agree well with those of the previous study^37^.

## Discussion

OCE benefits from OCT, providing the ability to measure biological tissue with micrometer spatial resolution and subnanometre displacement sensitivity. The static operation principle of SD-OCT contributes to its wide use in OCE. However, recent advancements in SS-OCT have proven its utility in OCE, especially with the advantages of enhanced imaging range, depth, and speed, increased SNR, and reduced phase washout. The phase stability of SS-OCT can be improved through optical and electrical optimization, but reducing the phase fluctuation in a conventional SS-OCT setup is fundamentally challenging, as the reference and sample signals travel through different optical paths. We have previously demonstrated a method to optimize the electrical components in an SS-OCT system to achieve high-phase stability^38^, and in this study, we employed a common-path configuration to further attain the peak performance of SS-OCE. This was accomplished by enhancing the SNR through averaging, minimizing the timing jitter by adjusting the electrical delay, and reducing the fluctuation of the OPD via the common-path configuration. The resulting SS-OCE_CP_ demonstrated a phase stability of 4.2 mrad, which was not only obtained in the system characterization but also achieved during the experiments. In addition to the 40-fold improvement in the phase stability compared to that of SS-OCE_COV_, SS-OCE_CP_ showed a 3-dB improvement in the imaging SNR, all without the need for external stabilization or extravagant post-processing.

The phase performance of our SS-OCE_CP_ was first validated using a tissue-mimicking silicone phantom. The improved phase stability was reflected by the more pronounced displacement boundary of the elastic wave than that of SS-OCE_COV_. We repeated the experiment using an in vivo rabbit corneal model to further demonstrate the improved capability of SS-OCE_CP_ to retrieve precise phase information for elasticity quantification. The results of the IOP experiment are also supported by reported studies^37^. Additionally, the improved displacement sensitivity provided by SS-OCE_CP_ can reduce the minimum ARF required to induce a detectable tissue displacement, which is essential for future clinical translation.

Although we have demonstrated the superior performance of SS-OCE_CP_, there are a few design improvements that can be implemented. First, in the proposed optical configuration, the reference signals are generated by the front and back surfaces of the 3-mm wedged window. To achieve the optimal SNR, the travel distance of the detection beam from the scan lens to the first surface of the window must be constant to provide a uniform back-reflected signal; even a slight deviation will cause a variation in the OPD and back-reflected power during scanning, resulting in a reduced SNR. For applications that require scanning of a larger area, a scan lens that provides a larger field of view should be used. Furthermore, for three-dimensional OCE, a 4 F optical corrector can be incorporated into the scanning design to ensure a constant entrance pupil and OPD.

In addition, we previously demonstrated a confocal alignment between the OCT detection beam and the excitation via a coaxial configuration for high excitation efficiency^15,32^. A confocal/coaxial setup can improve the ease of use and reduce the form factor of the sample arm, further facilitating the translation of this technology to clinical use. In our current SS-OCE_CP_ design, the wedged window prevents this confocal setup, as the ARF will be attenuated by the window. Alternatively, the wedged window can be fabricated using an optically and acoustically transparent material, such as Pebax® and low-density polyethylene^39,40^. With this window, a ring-shaped ultrasound transducer can be inserted between the scan lens and the window for excitation, allowing the OCT detection beam to travel through the center of the transducer for imaging.

Finally, the light source of the SS-OCE_CP_ is a vertical-cavity surface-emitting laser with an internal MZI that provides a k-clock signal at ~400 MHz, allowing for an imaging range of ~11 mm in standard OCT or ~5.5 mm when utilizing the proposed averaging method that enhances the SNR by 3 dB. While a 5.5-mm imaging range may be sufficient in many applications, a long imaging range can be achieved through doubling the k-clock frequency by building an external circuit or through the dual edge sampling provided by certain waveform digitizers. A custom-built external k-clock can also be considered. Alternatively, an acousto-optic modulator can be incorporated to shift the frequency of the interferometer signal to take advantage of the full bandwidth of the frequency^41^. With respect to achieving a better penetration depth, swept-source lasers with a longer center wavelength, such as 1.7 μm, can be utilized^42^.

In summary, we have reported the first OCE system based on SS-OCT with a common-path configuration. A phase stability of 4.2 mrad was obtained, and the feasibility and performance of our SS-OCE_CP_ were tested and validated using a phantom and in vivo rabbit model. This highly phase-stable system can quantify displacement in the subnanometre range, and we believe that it has great potential in other applications that require precision phase measurement, such as flowmetry^11^, vibrometry^43^, and molecular imaging^44^.

## Materials and methods

### System setup

**Fig. 7:**
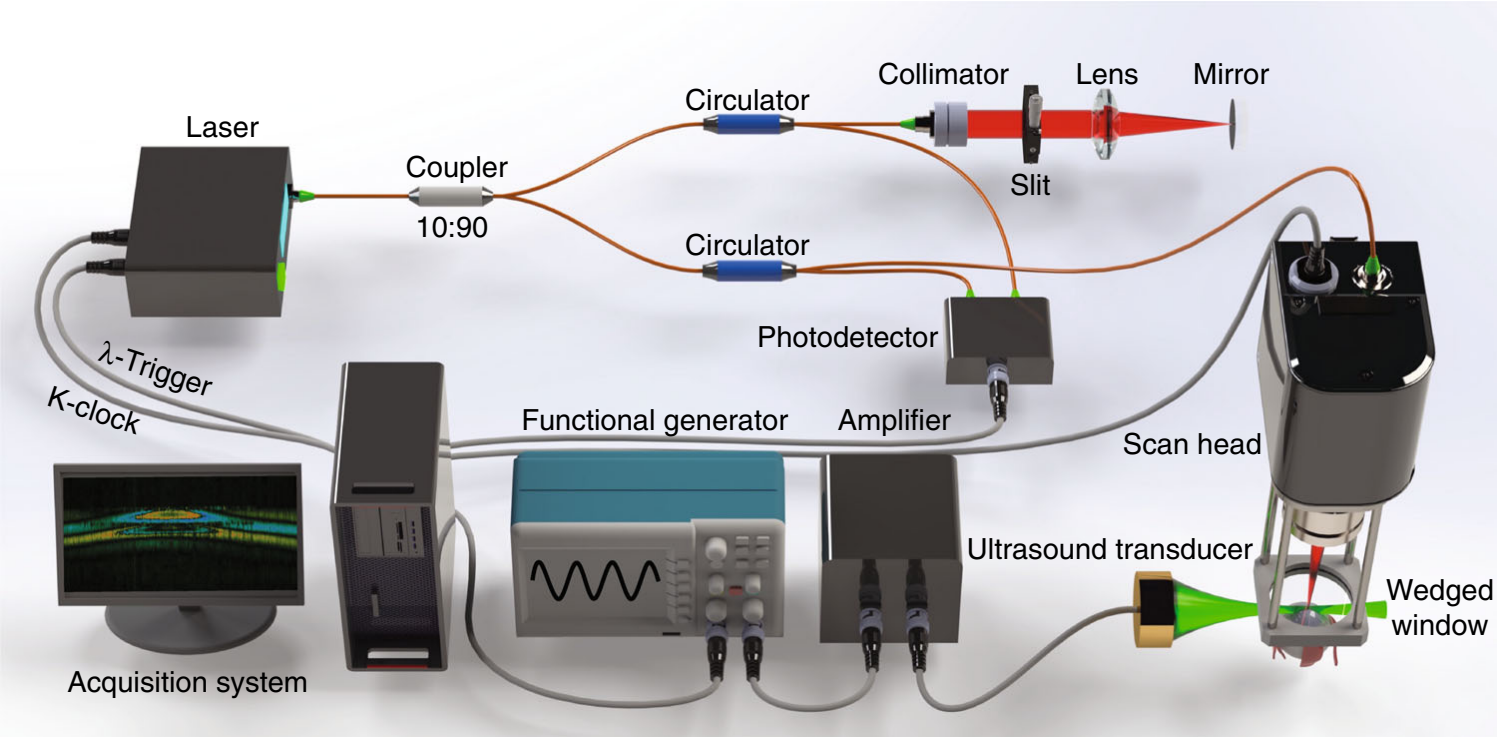
Schematics of the SS-OCE_CP_ system

**Fig. 8:**
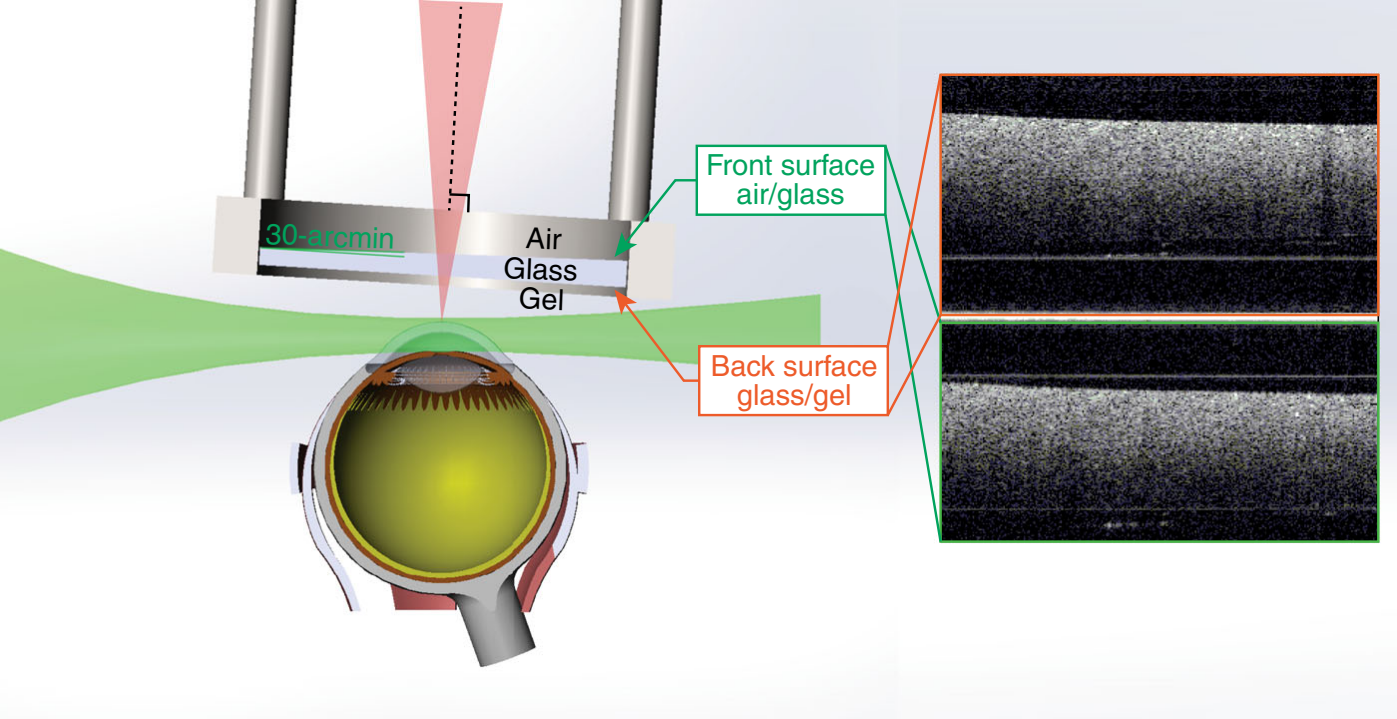
Schematics of the 30-arcmin wedged window

An SS-OCE_CP_ system was designed and constructed. The swept laser (SL1310V1-10048, Thorlabs, Inc., NJ) has a repetition rate of 100 kHz, a center wavelength of 1310 nm, and a bandwidth of 100 nm. The system has an imaging range of 11 mm, which makes the generation of two OCT images in different frequency domains possible. The output light from the laser source is split by a 90:10 optical fiber coupler, with 90% of the light propagating to the sample through a circulator, a collimator, an objective scan lens (LSM04, Thorlabs, Inc., NJ), and a wedged window, as shown in Fig. 7. The common-path configuration is achieved through the 30-arcmin wedged window. In contrast to a flat window, the 30-arcmin angle on the front surface effectively reduces the autocorrelation fringe patterns generated within the window cavity, as shown in Fig. 8. In this setup, two interfaces exist: the air-glass front surface and the glass-gel back surface. The indices of refraction of the air, glass (the wedged window), and ultrasound gel are 1.0, 1.5, and 1.34, respectively. The back surface of the wedged window is perpendicular to the OCT scanning beam, while the front surface is shifted by 30 arcmin from the normal direction. The differences in the refractive indices and in the corresponding incident angles contribute to the discrepancy in reflectance of the two surfaces, but this can be compensated for by adjusting the position of the wedged window relative to the scan lens because the collected power of the focusing back-reflected light is spatially dependent (Supplementary Material, Fig. S4). As such, two back-reflected reference signals of similar power interfere with the backscattered sample signal to generate two distinct fringes corresponding to the top and bottom images. The two interferences are then delivered to one channel of the balanced photodetector. The wedged window has a thickness of 3 mm, which corresponds to a 3.4-mm axial separation of the generated OCT images calculated based on the ultrasound gel reflective index of 1.34. Additionally, the two images can be averaged to enhance the SNR and minimize speckles. To enable balanced detection, the remaining 10% of the light propagates through a compensation arm consisting of a circulator, a collimator, an adjustable slit and a mirror. The back-reflected light from the compensation arm is detected by the second channel of the balanced photodetector to offset the DC component of the generated interference fringes. A balanced photodetector with a bandwidth from 30 kHz to 1.6 GHz was selected to minimize timing jitters to enhance the phase stability and to enable a long imaging range for retrieving the two OCT interference fringes from different frequency domains^14^.

For the displacement excitation, a custom-built 4.5-MHz ultrasound transducer with a focal length of 35 mm is placed approximately orthogonal to the scan lens. A function generator is synchronized with the λ-trigger signal to generate a 4.5-MHz sine wave (duration: 200 μs) that is then amplified by approximately 42 dB to drive the ultrasound transducer for tissue excitation. The space between the wedged window, the ultrasound, and the sample is filled with ultrasound gel to couple the ARF.

For comparison, SS-OCE_CP_ can be quickly converted to SS-OCE_COV_ during the experiments while maintaining the exact location of the sample and the ultrasound transducer relative to the OCT detection beam (Supplementary Material, Fig. S5).

### Data acquisition, synchronization, and signal processing

Two timing signals produced by the swept-source laser are utilized for data acquisition and synchronization. The k-clock signal generated by the internal MZI provides a timing of equal wavenumber spacings, and the λ-trigger signal is produced by the internal FBG to give a temporal mark for each wavelength sweeping. In conventional SS-OCT or SS-OCE imaging systems, a series of OCT signal points is digitized starting at the edge of the λ-trigger, while the signal digitization is clocked by the k-clock signal for k-linear signal sampling. However, in this mode of operation, random timing errors of the signal edges can be magnified to significant timing fluctuations between the k-clock and λ-trigger through a process termed edge collision^38^. To achieve the best stability, the signal delays are adjusted to the optimal values for the k-clock, λ-trigger and OCE signals. A previous study on the phase stability with the same type of swept lasers suggested that a very high stability could be obtained using this method^38^.

The propagation velocity of the elastic wave provides a direct measurement of the biomechanical property, as pre-calibration is not necessary to convert the displacement to elasticity. To visualize the elastic wave propagation, an M-B scanning protocol is utilized to induce and detect the displacement. At each lateral position, 500 A-lines are acquired to record the phase change over time (M-mode). The ultrasound transducer is excited after a trigger delay of 1 ms to generate an ARF with a duration of 200 μs for each M-mode acquisition. After one M-mode acquisition, the galvanometer scanner moves the detection beam to the next lateral position, and the same step is repeated. A total of 3000 M-mode images are acquired for each dataset to convert to B-mode images. The scanning protocol is summarized in Fig. S6 in the Supplementary Material. The phase-resolved Doppler algorithm is applied to extract the temporal phase information. With a time interval of 50 μs, inter-A-line analysis is performed to obtain time-lapse Doppler OCT B-scans and spatiotemporal Doppler OCT images. Young’s modulus can then be calculated by determining the propagation velocity using the spatiotemporal Doppler images. Because the different boundary conditions yield different propagation modes of the elastic waves, a specific equation is used to calculate the elasticity based on the sample types^8,45^. In our experiment, Young’s modulus, *E*, is calculated based on the Rayleigh wave velocity, *V*_R_, and Lamb wave velocity, *V*_L_, for the tissue-mimicking phantom and rabbit cornea, respectively. The data processing steps and key equations for elasticity calculation are detailed in Fig. S7 in the Supplementary Material.

### Phantom preparation

To fabricate the silicone-based phantom that mimics tissue biomechanical properties, 2 g of titanium dioxide was added to every 100 g of silicone rubber base (P4—Part B, Eager Polymers, Inc., IL) and mixed using an ultrasonic cleaner. Then, 1 part of the silicone rubber base with well-mixed titanium dioxide was added to 16 parts of silicone activator (P4—Part A, Eager Polymers, Inc., IL). After the base was gently mixed with the activator using a stirring rod, the mixture was placed into a vacuum chamber until all the air bubbles trapped inside the mixture were removed. The mixture was then poured into a container for molding and cured for 24 h. The final phantom had dimensions of 40 mm × 40 mm × 10 mm.

### **I**n vivo rabbit experiment preparation

To induce the initial anesthesia, the rabbit (New Zealand white rabbit, male, ~3 kg) was administered a ketamine-xylazine mixture (35 mg/kg and 5 mg/kg, respectively) subcutaneously. Two drops of 2.5% proparacaine hydrochloride were applied topically for local anesthesia. After conforming to the proper depth of anesthesia, the rabbit was placed onto a stage for position adjustment. The rabbit’s eye was covered with sterile ultrasound gel to provide a conductive medium between the eye and the acoustic wave for the IOP experiment. To obtain a higher IOP, the rabbit eye was carefully proptosed, and a sterile latex drape with an aperture was put through the globe to maintain proptosis. Eye proptosis in rabbits typically increases the IOP to ~50 mmHg. All procedures were reviewed and approved by the Institutional Animal Care and Use Committee at the University of California, Irvine, under protocol #AUP-19-042.

## Supplementary information

Supplementary Information
