# Supplementary material for: Ultrahigh-sensitive optical coherence elastography
Source: Light Sci Appl. 2020 Apr 13;9:58. doi: 10.1038/s41377-020-0297-9 (PMC7154028; doi:10.1038/s41377-020-0297-9)

**Ultrahigh-Sensitive Optical Coherence Elastography**

**Yan Li^1,2^, Sucbei Moon^1,3^, Jason J. Chen^1,2^, Zhikai Zhu^1,2^, Zhongping Chen^1,2,*^**

^1^Beckman Laser Institute, University of California, Irvine, Irvine, CA, 92612, USA

^2^Department of Biomedical Engineering, University of California, Irvine, Irvine, CA,92617, USA

^3^Department of Physics, Kookmin University, Seoul 02707, South Korea

* [z2chen@uci.edu](mailto:z2chen@uci.edu)

**Figure S1. Velocity and Young’s modulus quantification**

Comparison of (a) elastic wave velocities and (b) Young’s moduli in the tissue mimicking phantom and cornea obtained from SS-OCE_COV_ and SS-OCE_CP_. The measurements were taken from a phantom and a rabbit and were repeated 5 times in each case.


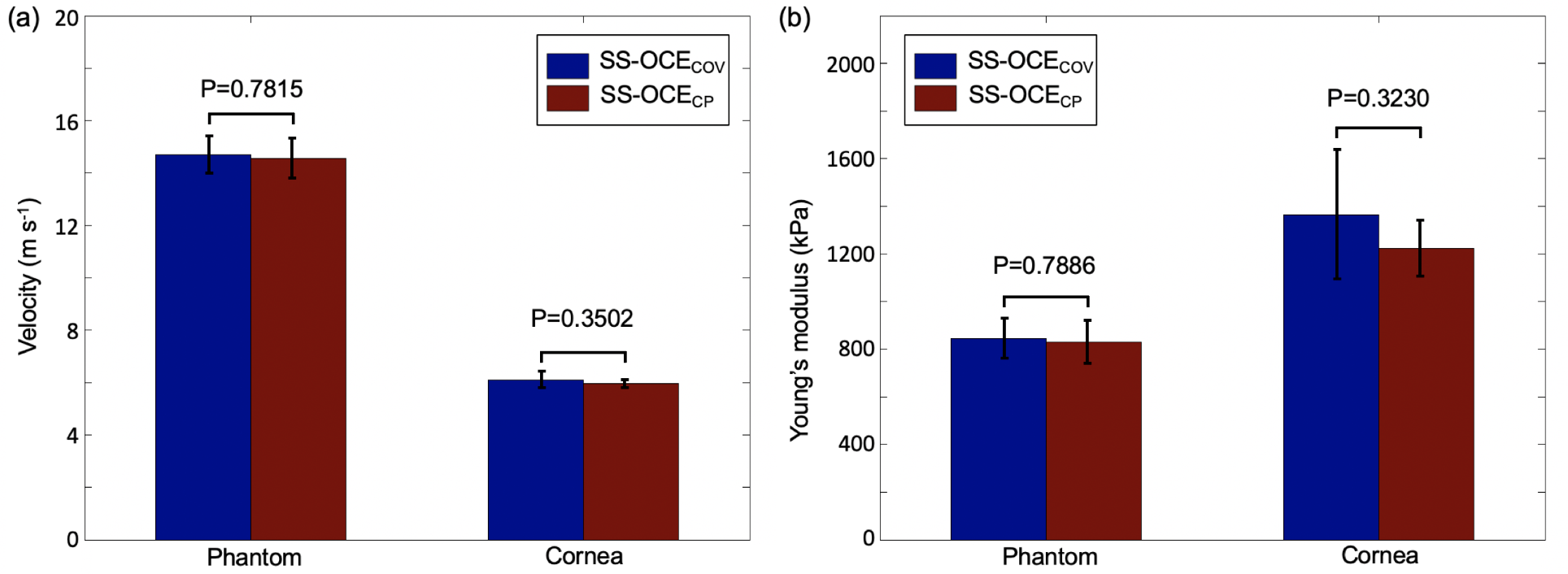


**Figure S2. *In vivo* IOP experiment in a rabbit model**

(a-d) Time-lapse Doppler OCT B-scans of the normal cornea with normal IOP. (e-h) Time-lapse Doppler OCT B-scans of the normal cornea with high IOP. (i) and (j) Spatiotemporal Doppler OCT images with normal and high IOP, respectively.

**
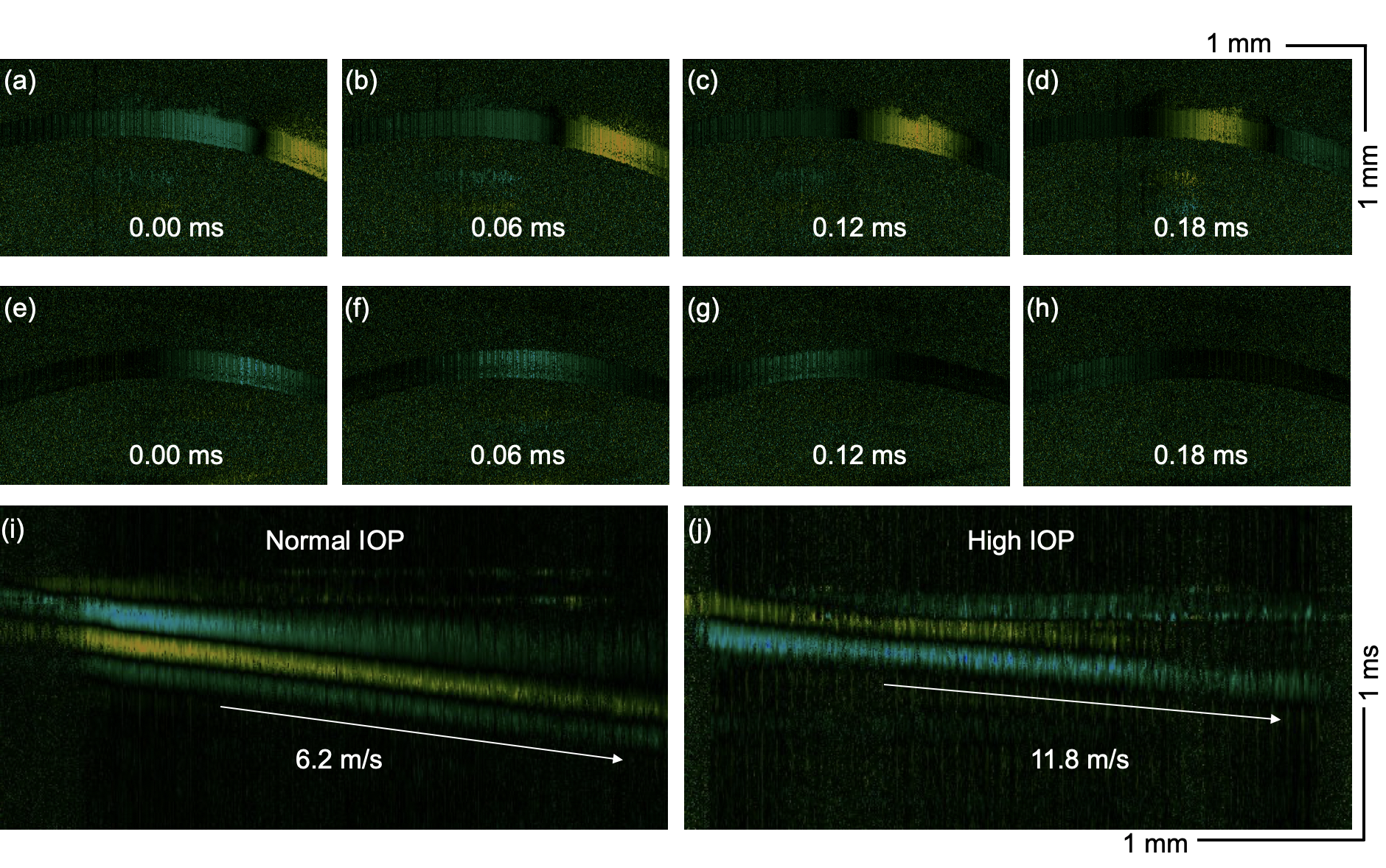
**

**Figure S3. Velocity and Young’s modulus of cornea with different IOP from SS-OCE_CP_**

The measurements were taken from one rabbit and repeated 5 times in each case.


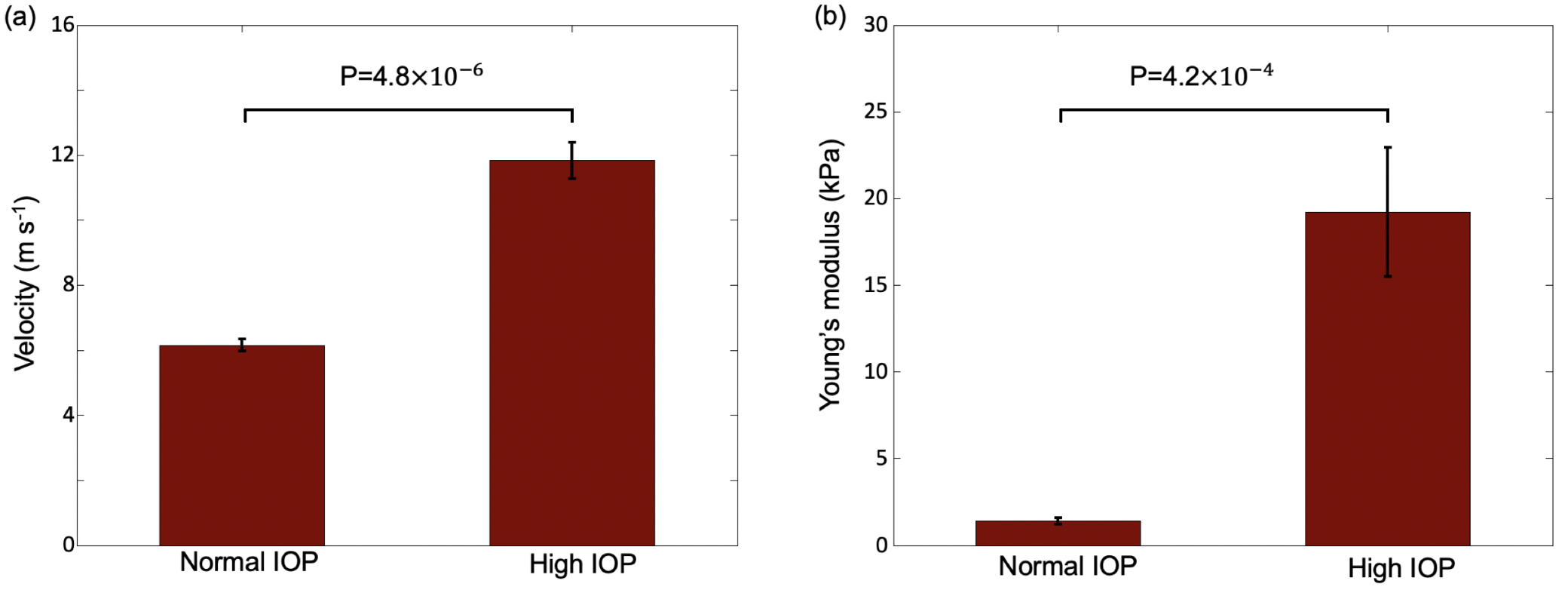


**Figure S4. Measured back-reflected power of a flat mirror vs. distance from focal point**

Assuming unpolarized light, the reflectance,$R$, of an interface can be calculated using the Fresnel equations:

|  | $R=\left[ \frac{n_{i}\cos\left( \theta_{i} \right)-n_{t}\cos\left( \theta_{t} \right)}{n_{i}\cos\left( \theta_{i} \right)+n_{t}\cos\left( \theta_{t} \right)} \right]^{2}$ | $(1)$ |
| --- | --- | --- |

where $n_{i}$ is the incident refractive index, $n_{t}$ is the transmitted refractive index, $\theta_{i}$ is the incident angle, and $\theta_{t}$ is the transmitted angle. The refractive indices and the corresponding reflection coefficients are reported in Table 1:

| Interface | $\boldsymbol{n}_{\boldsymbol{i}}$ | $\boldsymbol{n}_{\boldsymbol{t}}$ | $\boldsymbol{\theta}_{\boldsymbol{i}}$ | $\boldsymbol{\theta}_{\boldsymbol{t}}$ | $\boldsymbol{R}$ |
| --- | --- | --- | --- | --- | --- |
| Air-glass | 1.0 | 1.50 | 0.5° | 0.33° | 4% |
| Glass-gel | 1.50 | 1.34 | 0.33° | 0.37° | 0.32% |

We note that $R$ of the air-glass interface is approximately 10 times greater than that of the glass-gel interface. In order to equalize the reflective power from the two interfaces, we offset the difference in the collection efficiency of back-reflected power through defocusing. In Figure S5, we report the measured back-reflected power through the scan lens from a flat mirror as a function of distance from the focal point, where distance = 0 represents the focal point of the scan lens. To balance the collected back-reflected power from the two interfaces, we placed the wedged window such that its air-glass and glass-gel interfaces are positioned at “A” and “B” of Figure S5, respectively. Because the collection efficiency of back-reflected power at A is about 10 times less than that at B, we can expect the signals collected from the two interferences are approximately balanced.


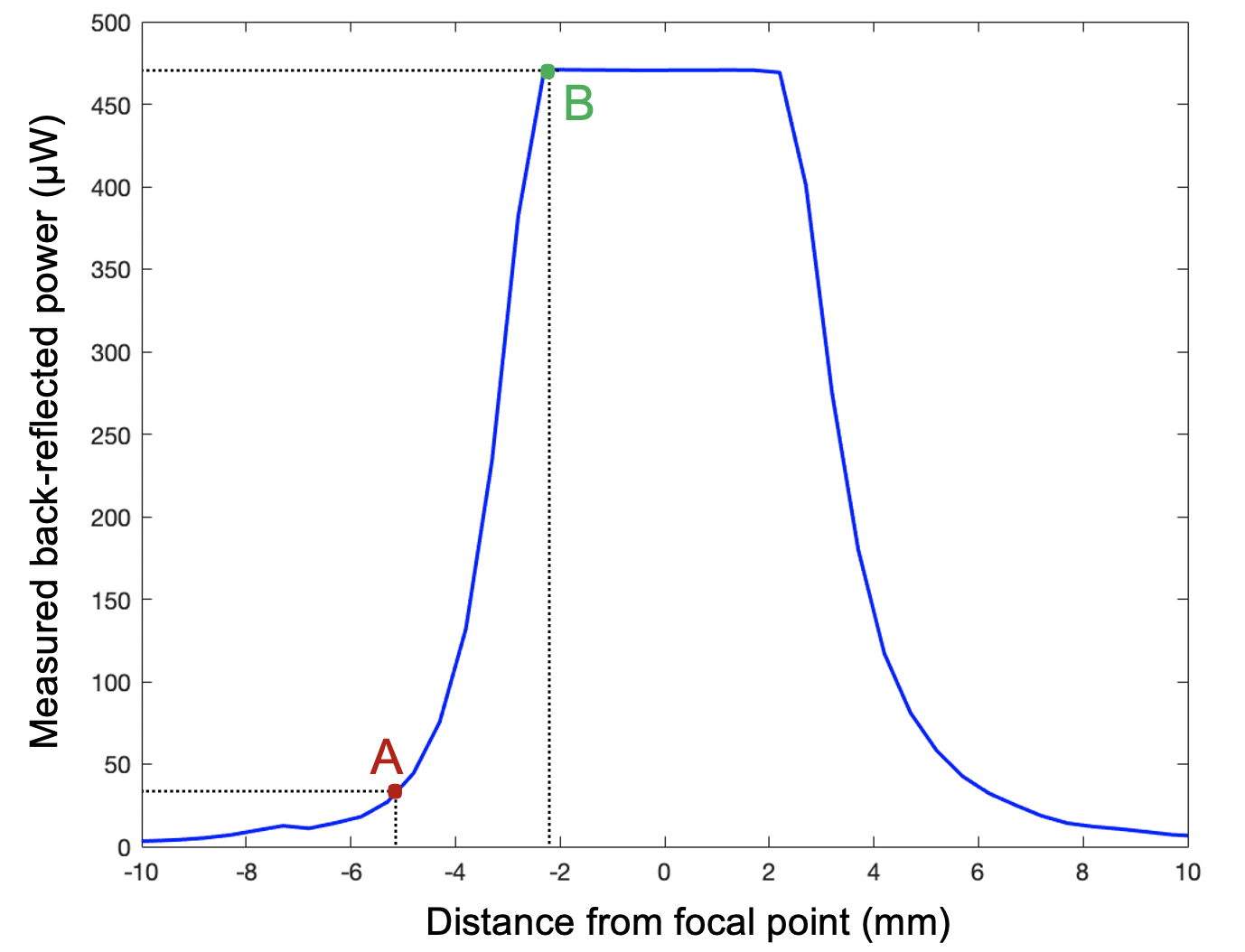


**Figure S5. SS-OCE_COV_ setup**

SS-OCE_CP_ can be conveniently converted to SS-OCE_COV_ through two modifications, as shown in Figure S2(a). A 50:50 coupler was inserted between the balanced photodetector and Port 3 of the circulators, in which the back-reflected light from the reference arm and the back-scattered light from the sample arm interfere to generate the signal. Additionally, a small amount of water was added onto the front surface of the window, as shown in Figure S2(b). Because the scan head was tilted by a small angle, the water surface is oblique with the OCT beam, which reduces the reflection from the water and window effectively, hence minimizing the autocorrection. The use of a balanced photodetector can remove the residual autocorrection.


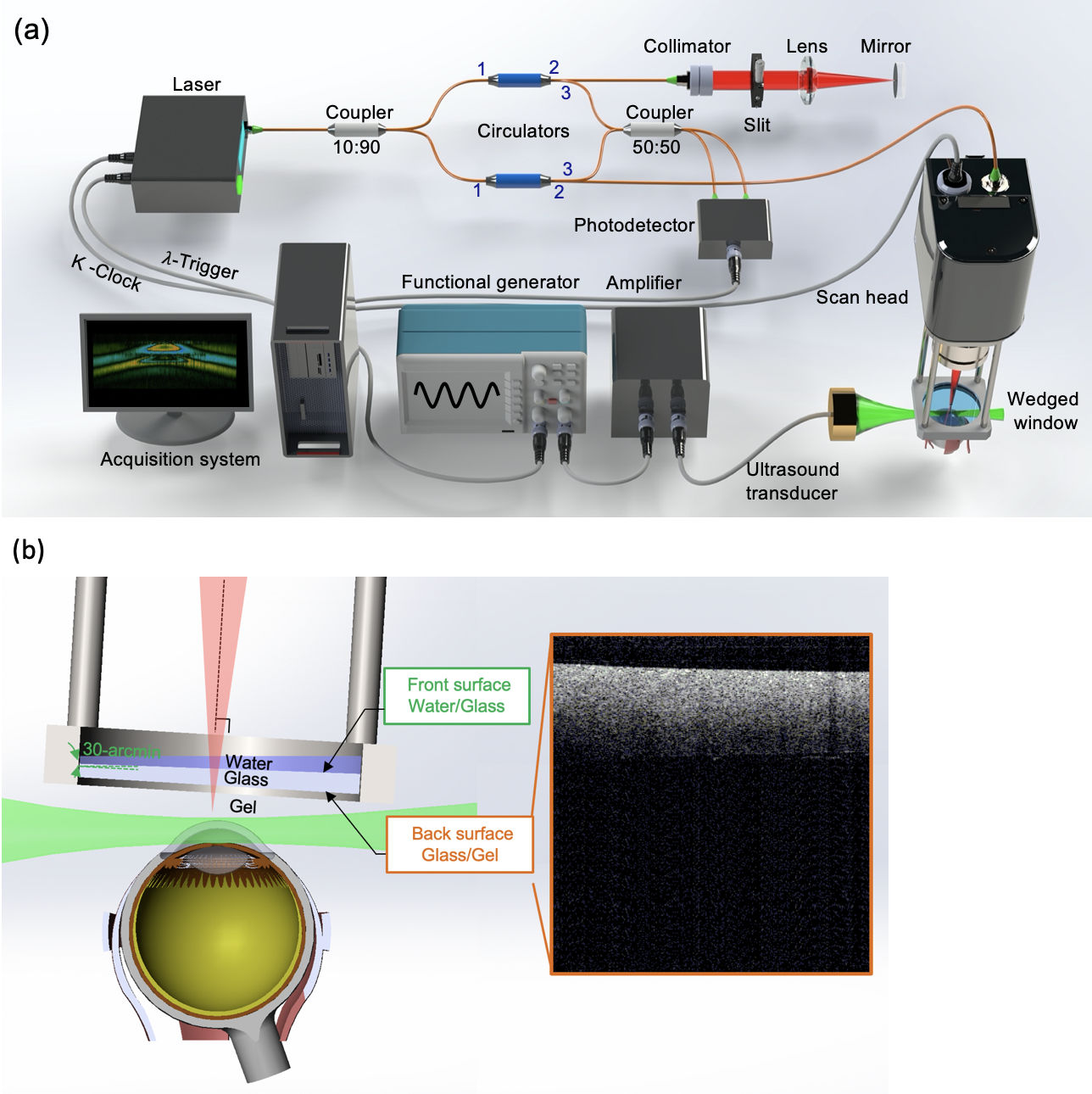


**Figure S6. Scanning scheme of the ARF-OCE system**

(a) Trigger signals from the laser is used to synchronize data acquisition. One image consists of 500 A-lines. (b) A synchronized gate signal with a duration of 200 $\mu s$ is used for ARF excitation. (c) Signal for the x-axis galvanometer scanner to employ M-B mode scanning. (d) and (f) OCT image obtained through M-B mode protocol. (e) and (g) OCE images obtained through M-B mode protocol.

**
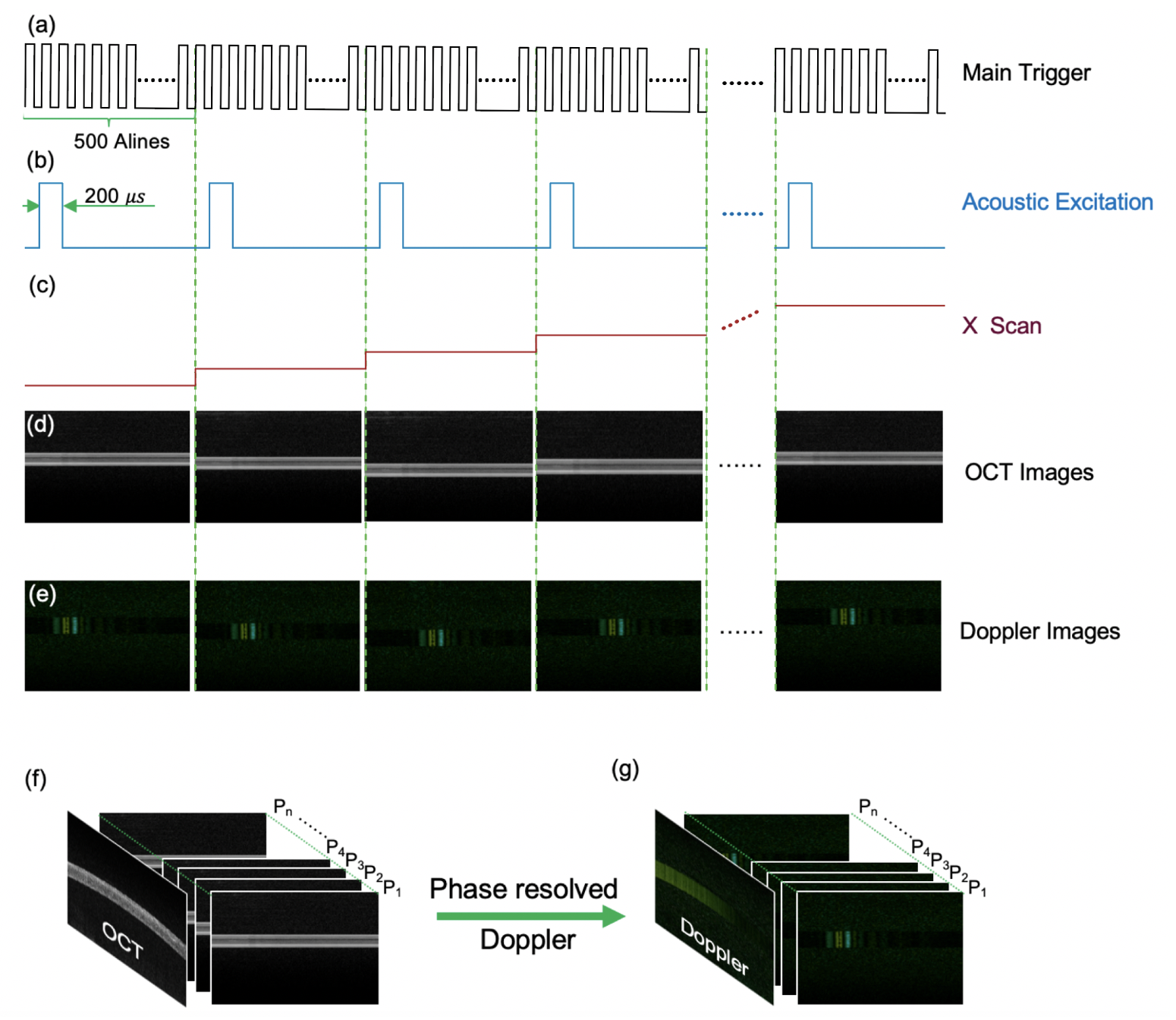
**

**Figure S7. Signal processing**

To calculate the Young’s modulus, we assumed a phantom density ($\rho$) of 1200 kg/m^3^, corneal density ($\rho$) of 1062 kg/m^3^, and Poisson’s ratio ($\nu$) of 0.5. $F_{m}$ : complex OCT signal*.* $F_{m+1}$ : adjacent complex OCT signal.$\nu:$Poisson’s ratio. $V_{R}:$detected wave velocity of the phantom. $V_{L}:$ detected wave velocity of the cornea. $f$: frequency of the Lamb wave. $h$: sample thickness.


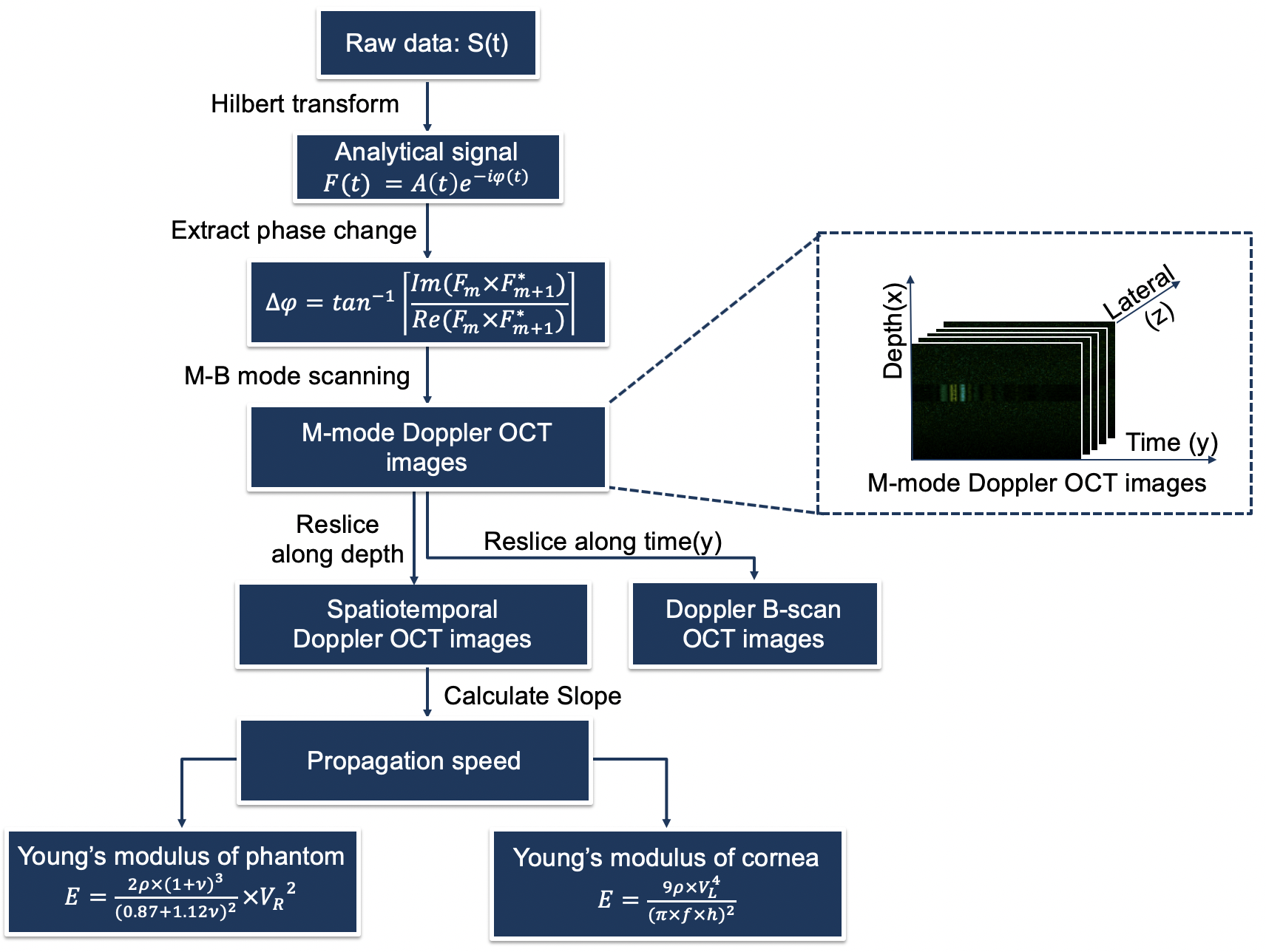

Supplement: Supplementary file 1 — Supplementary Information [file 41377_2020_297_MOESM1_ESM.docx]
